# Cholesteric liquid crystal biosensor platform with image analysis for rapid detection of COVID-19

**DOI:** 10.3389/fbioe.2023.1148446

**Published:** 2023-02-28

**Authors:** Ping-Yuan Lin, Yi-Wei Chung, Er-Yuan Chuang, Yen-Chieh Wang, Min-Chih Lai, Yen-Chin Hsu, Chun-Che Lin, Yu-Cheng Hsiao

**Affiliations:** ^1^ Graduate Institute of Biomedical Opto mechatronics, College of Biomedical Engineering, Taipei Medical University, Taipei, Taiwan; ^2^ Division of Cardiology, Department of Internal Medicine, Shuang Ho Hospital, Taipei Medical University, New Taipei City, Taiwan; ^3^ Taipei Heart Institute, Taipei Medical University, Taipei, Taiwan; ^4^ Cardiovascular Research Center, Taipei Medical University Hospital, Taipei, Taiwan; ^5^ Division of Cardiology, Department of Internal Medicine, School of Medicine, College of Medicine, Taipei Medical University, Taipei, Taiwan; ^6^ Graduate Institute of Biomedical Materials and Tissue Engineering, College of Biomedical Engineering, Taipei Medical University, Taipei, Taiwan; ^7^ School of Biomedical Engineering, Taipei Medical University, Taipei, Taiwan; ^8^ School of Nutrition and Health Sciences, Taipei Medical University, Taipei, Taiwan; ^9^ Institute of Organic and Polymeric Materials, National Taipei University of Technology, Taipei, Taiwan; ^10^ Cell Physiology and Molecular Image Research Center, Wan Fang Hospital, Taipei Medical University, Taipei, Taiwan; ^11^ Stanford Byers Center for Bio Design, Stanford, CA, United States

**Keywords:** COVID-19, cholesteric liquid crystal, biosensor, diagnosis, spike protein

## Abstract

Rapid and low-cost diagnosis of coronavirus disease 2019 (COVID-19) is essential to identify infected subjects, particularly asymptomatic cases, primarily to arrest the spread of the disease through local transmission. Antibody-based chromatographic serological tests, as an alternative to the RT-PCR technique, offer only limited help due to high false positives. We propose to exploit our cholesteric liquid crystal biosensor platform for one-step, wash-free, rapid detection of the severe acute respiratory syndrome coronavirus-2 (SARS-CoV-2) virus directly with minimal sample pre-processing. As mentioned above, cholesteric liquid crystals are an effective and innovative approach to healthcare as a rapid test for the diagnosis of COVID-19 and other diseases.

## 1 Introduction

The coronavirus disease 2019 (COVID-19) outbreak, caused by severe acute respiratory syndrome coronavirus-2 (SARS-CoV-2), has had major impacts globally since the first case was reported in China in December 2019 (Azzi et al., 2020). The high infectivity and rapid spread of the virus have posed serious threats across the globe, as witnessed by the steep rise in mortality rates in the last 4 months. To tackle this problem, the transmission of the virus at the community level can be reduced/prevented through social distancing and other measures including personal hygiene. Further, mass screening to identify infected people (with or without symptoms) and their isolation and appropriate treatment have shown positive progress in breaking the chain of community transmission. Currently, the reverse-transcription polymerase chain reaction (RT-PCR) technique is widely used to detect SARS-CoV-2, which usually takes a few hours for analysis. However, its wide-scale deployment in resource-constrained settings is limited as it requires expensive equipment and trained personnel. Considering that a large population with suspected or confirmed COVID-19 exists, there is an urgent need for a rapid diagnostic tool (Braz-Silva et al., 2021).

For rapid detection of COVID-19, typically antibody-based diagnostic assays are preferred due to their convenient field application (Danny et al., 2020). Current strategies are based on the serological determination of neutralizing antibodies produced in the host as a defense response against SARS-CoV-2. However, delayed immune responses along with large variations in the serum immunoglobulin M (IgM)/IgG antibody levels in infected persons may pose a risk of false-positive/negative results (Divagar and Sai, 2018). An efficient alternate bioanalytical approach could be the specific detection of the nucleocapsid (N) protein, an important protein of the SARS-CoV-2 virus.

Recently, liquid crystal (LC) biosensors for disease diagnosis were successfully demonstrated (Han and Martin, 2011; Pan et al., 2013; Dischinger et al., 2017; Su et al., 2019; Chuang et al., 2021a; Lu et al., 2021a; Lu et al., 2021b; Duong and Jang, 2021; Huang et al., 2021). Biomolecules can redirect LC molecules and cause intensity changes in the light signal (Burnouf et al., 2019). Redirection of LCs causes a sensitive response to changes in the optical signal. An earlier study combined LC with a microfluidic device to detect bovine serum albumin (BSA) (Fan et al., 2020; Chen et al., 2021). Compared to nematic LCs, cholesteric LCs (CLCs) possess unique optical viewing angles: Bragg reflection, bistability, and material flexibility (Hsiao et al., 2011a; Hsiao et al., 2011b; Hsiao et al., 2011c; Hsiao et al., 2013). The first CLC biosensor with high sensitivity of color specificity (with a limit of detection (LOD) of 1 fg/mL) for detecting BSA was developed in 2015 (Hsiao et al., 2015; Luh et al., 2022). The real sample was also test in CLC biosensor with worse LOD (Chuang et al., 2021b; Luh et al., 2022). However, to our knowledge, this is the first study to use color-specific CLC biosensors as an innovative medical approach for the early detection of COVID-19.

**FIGURE 1:**
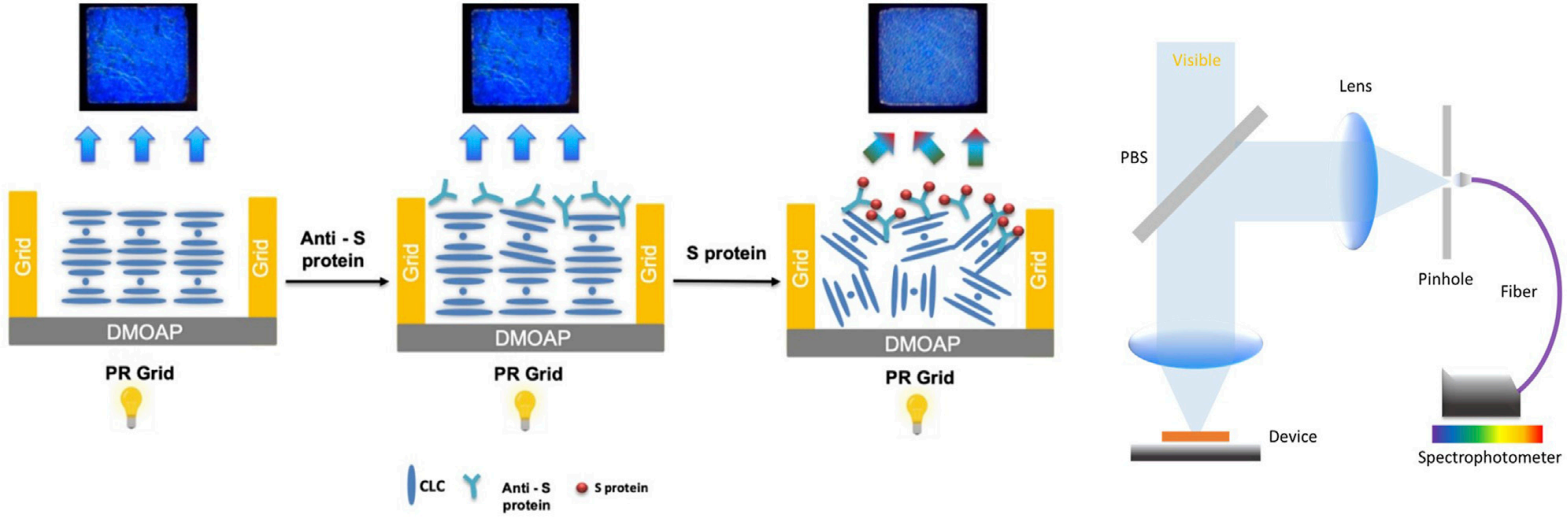
Schematic diagram of the detection mechanism of the cholesterol liquid crystal chip.

In this study, we propose a rapid COVID-19 CLC biosensor. The assay was performed using the behavior between glass substrate pairs coated with an antigen/antibody and CLCs. This rapid CLC-based biosensing chip greatly differs from typical biosensors. The SARS-CoV-2 spike protein antigen/antibody can be measured with the naked eye. Alignment layers of N, N-dimethyl-n-octadecyl-3-aminopropyltrimethoxysilyl chloride (DMOAP) were used to measure spike protein concentrations. A schematic diagram of this COVID-19 rapid detection CLC sensor is shown in Figure 1. The innovation of this work is that a point-of-care device for home use is proposed for the first time as a rapid test for COVID-19.

## 2 Materials and methods

### 2.1 Preparation of the CLCs

In this experiment, a mixture of four different nematic LCs, 5CB, 7CB, 8OCB, and 5CT, was synthesized in a specific ratio, and the LCs were mixed with R5011, a spin-parallel molecule (3.03%), with rotational force. The LCs were then placed at room temperature and cooled to the LC state, resulting in CLCs with a wavelength of approximately 450 nm that reflects blue light.

### 2.2 Photoresistive grid trim DMOAP alignment layer

DMOAP is a long-chain alkyl compound, which is often used as a vertical coordinating agent in LC experiments, and after hydrolysis, DMOAP can be plated on the photoresist grid to form a thin film. The DMOAP structure is C18H37, and the long-chain alkyl structure allows this molecule to enter the photoresist grid (the photoresist grid was provided by Prof. Chih-Hsin Chen’s laboratory, Department of Chemistry, Tamkang University, Taipei, Taiwan). The CLCs could be successfully filled into photoresist grids without soaking in phosphate-buffered saline (PBS) water solution by plating DMOAP onto the photoresist grids. First, the photo-resistive grids were cleaned with deionized (DI) water, and then the grids were soaked in a 0.1% DMOAP dye pot for 15 min. Next, the grids were taken out, cleaned twice more with DI water, and then nitrogen was used to blow off the excess DMOAP solution. Finally, the photoresistive grids were placed in an oven to dry at 100°C, and the DMOAP-coated photoresistive grids were finished.

### 2.3 Fabrication of the CLC detection chip

First, the CLCs were heated to 80°C for 30 min to a fully transparent liquid state and then placed at room temperature until the CLCs were restored to an LC state. CLCs at 0.1 µL were filled into a photoresist grid to a thickness of 8 μm, and the grid was rotated at 3,600 rpm for 1 min using a rotary applicator to evenly distribute the blue CLCs on the grid.

### 2.4 CLC detection system

The prepared CLC devices were placed at room temperature in a container of PBS-prepared spiked protein solution, changes in the optical state of the CLCs in different environments were observed using a polarizing microscope, and optical images were captured using a camera for spectral and image analyses.

## 3 Results and discussion

### 3.1 Mechanism of detection of CLC chips

We developed a system for detecting blue cholesterol-based LCs that employs a probe, an anti-spike protein (receptor binding domain; RBD), abbreviated Anti-Sp from Thermo fisher, that immunologically reacts with the antigen spike protein (RBD), abbreviated Sp from Thermo fisher. This CLC detection system allows for the observation, quantification, and analysis of CLCs. This CLC detection system can observe, quantify, and analyze optical changes in CLCs. The blue cholesterol-based LC detection system is depicted schematically in Figure 1. Since we used the Anti-Sp, an antibody with high affinity and specificity for the Sp, the antibody can effectively capture the Sp. Therefore, we added the Sp to the CLC detection system, and the protein was only at the interface between the LCs and the aqueous solution. When the antigen Sp was added to the PBS aqueous solution, the antibody bound to the antigen formed an immune complex with a molecular weight sufficient to cause disturbance in the arrangement of the LC molecules. When the CLC formed a focal cone (FC) in the air environment, they formed a flat state in the water environment. The arrangement of the CLC molecules was disrupted when the antibody successfully reacted with the antigen. The state of the CLC changed from flat to FC. The goal of detecting Sp can be achieved by changing the state of the CLCs.

### 3.2 Optical changes in the CLC sensor in different environments

**FIGURE 2:**
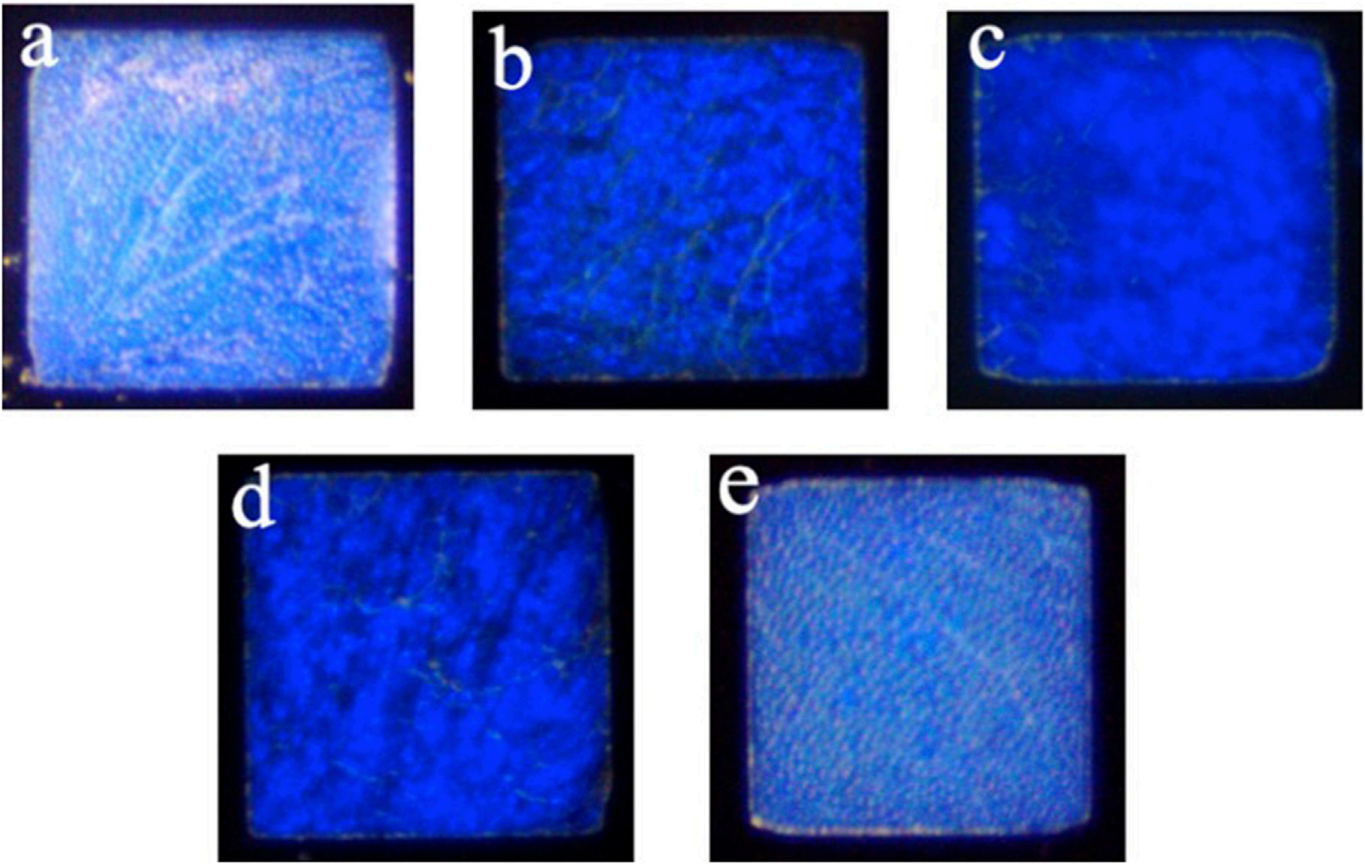
Optical images were taken by an orthogonal polarizing microscope under different environments of cholesteric liquid crystals. **(A)** Air environment, **(B)** PBS solution, **(C)** 0.001 μg/mL anti-spike protein (RBD), **(D)** 1 μg/mL spike protein (RBD), and **(E)** 0.001 μg/mL anti-spike protein (RBD) + 1 μg/mL spike protein (RBD).

The CLC detection system uses the bistable property of CLCs, the planar state when the CLCs are arranged neatly, and the FC state when the CLCs are arranged chaotically. The CLCs change their arrangement state according to different environments to produce different optical images and textures. Figure 2A is the FC state of CLCs when the image was captured by a polarizing microscope in the air environment; Figure 2B is the planar (P) state of CLCs when the CLCs were immersed in a PBS solution; Figure 2C is the P state of CLCs when the CLCs were immersed in a PBS solution containing 0.001 μg/mL Ant-Sp. Figure 2D is the CLC chip in an environment of a PBS solution containing 1 μg/mL SP which illustrates the P state; Figure 2E is the CLC chip in an environment of a PBS solution containing 0.001 μg/mL Anti-Sp and 1 μg/mL Sp which shows the FC state. According to the above optical image data, the CLCs exposed to air are in the FC state, but when immersed in a PBS aqueous environment, they appear in the P state. Adding the Anti-Sp or Sp alone did not affect the optical state of CLCs, but when Sp was added to the PBS solution containing the Anti-Sp, the optical state of the CLCs changed to the P state. When Sp was added to the PBS solution containing the Anti-Sp, the optical state of the CLCs changed to that of an FC state. From the above results, we know that when no antibodies are added to the solution, the CLCs do not react to the Sp and remain in the P state caused by the aqueous PBS environment.

### 3.3 Spectral analysis of the CLC detection system

In this paper, we used blue CLCs as the material. The wavelength of blue light is 400–500 nm in the visible light range, so we used a microfiber spectrometer to measure the penetration spectrum of the CLC chip in different environments.

**FIGURE 3:**
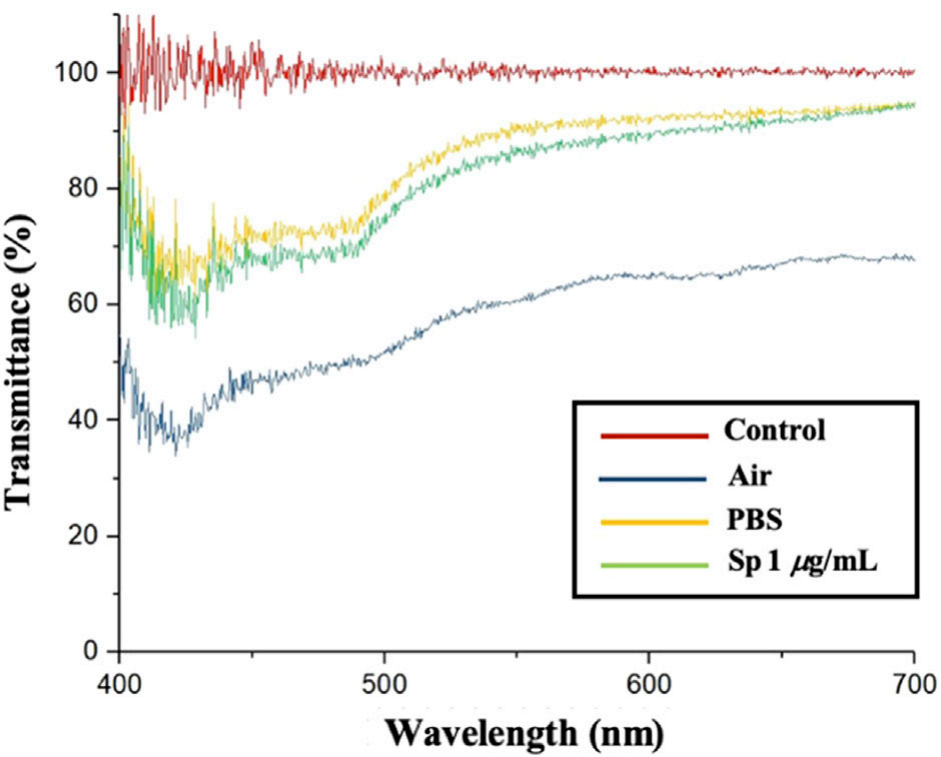
Cholesterol liquid crystal device penetration spectrum.

In Figure 3, the red line is the background value; the blue line is the CLC chip in an air environment; the yellow line is the CLC chip in a PBS aqueous solution, and the green line is the CLC chip in a 1 μg/mL Sp aqueous solution environment. In the air environment, the CLCs were in the FC state, the CLCs were arranged chaotically, the incident light was randomly scattered, and the penetration of the spectrum was lower without a complete waveform. In the PBS aqueous environment, the CLCs were in the P state, the CLCs were neatly arranged, the incident light reflected the blue wavelength with a complete wave band, and the penetration was higher. When only the antigen Sp was present, the CLCs did not react with the Sp in the P state because the Anti-Sp probe was not present. The penetration spectrum’s waveform and light penetration were similar to those in the PBS aqueous environment. According to the results, the CLCs produced different waveforms in the P and FC states, and the light penetration in the FC state was also much lower than that in the P state. Based on the above results, we could determine the state of the CLCs by the waveform and penetration in different environments.

### 3.4 Spectroscopic analysis of the CLC detection system

**FIGURE 4:**
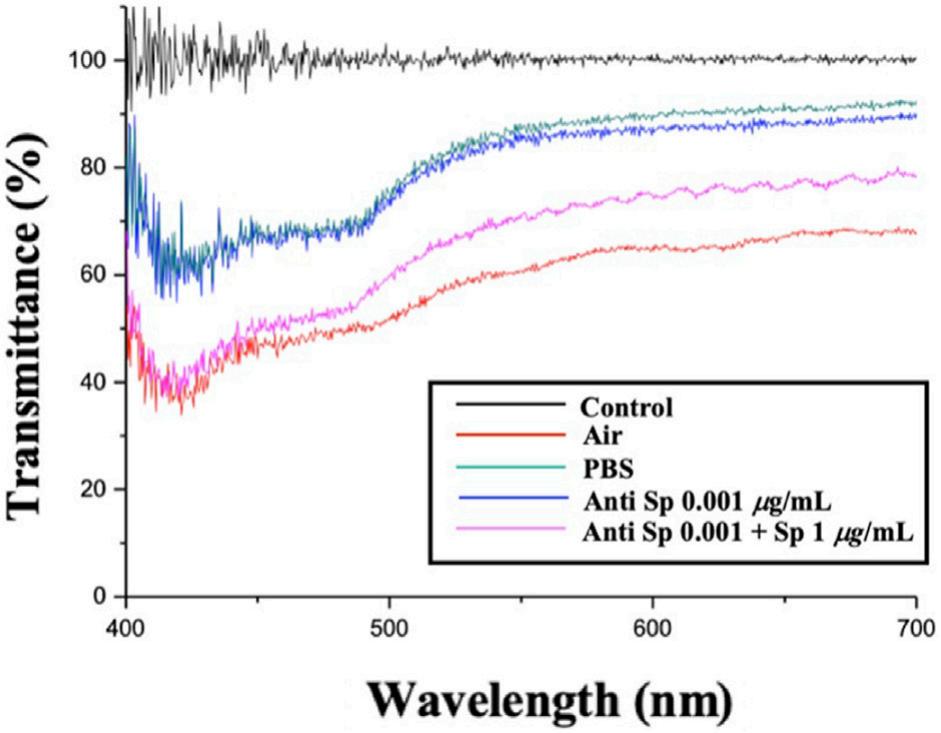
Spike protein in cholesterol liquid crystal chip detection by penetration spectroscopy.

This paragraph describes the measurement of the spectra of the CLC system with the addition of the Anti-Sp probe. Figure 4 shows the spectra of the CLC wafer in different environments: The black line is the background value; the red line is the CLC wafer in the air; the green line is the CLC wafer in an aqueous PBS solution, and the blue line is the CLC wafer in an aqueous solution with 0.001 μg/mL Anti-Sp. The pink line is the CLC wafer in an aqueous environment with 0.001 μg/mL Anti-Sp and 1 μg/mL Sp. In the air environment, the CLCs were in the FC state, and in the PBS aqueous environment, the CLCs appeared in the P state. When only the antibody was added, the CLCs appeared similar to the PBS aqueous environment and remained in the P state. When the antibody and antigen were added, because the antibody reacted with the antigen, the arrangement of the CLCs changed, and the CLCs changed from the P state to the FC state. It was established that the immune response can affect the optical state of the CLCs.

### 3.5 Detection sensitivity of CLC sensor chip

**FIGURE 5:**
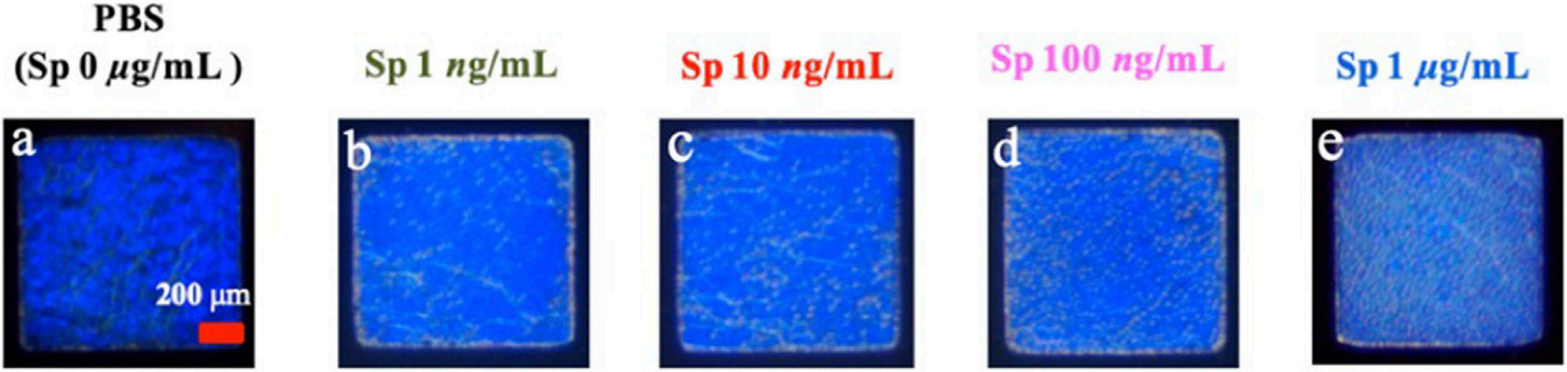
Optical images of cholesterol liquid crystals at **(A)** 0 ng/mL, **(B)** 1 ng/mL, **(C)** 10 ng/mL, **(D)** 100 ng/mL, and **(E)** 1 μg/mL concentration of the spike protein (RBD) in an aqueous solution.

**FIGURE 6:**
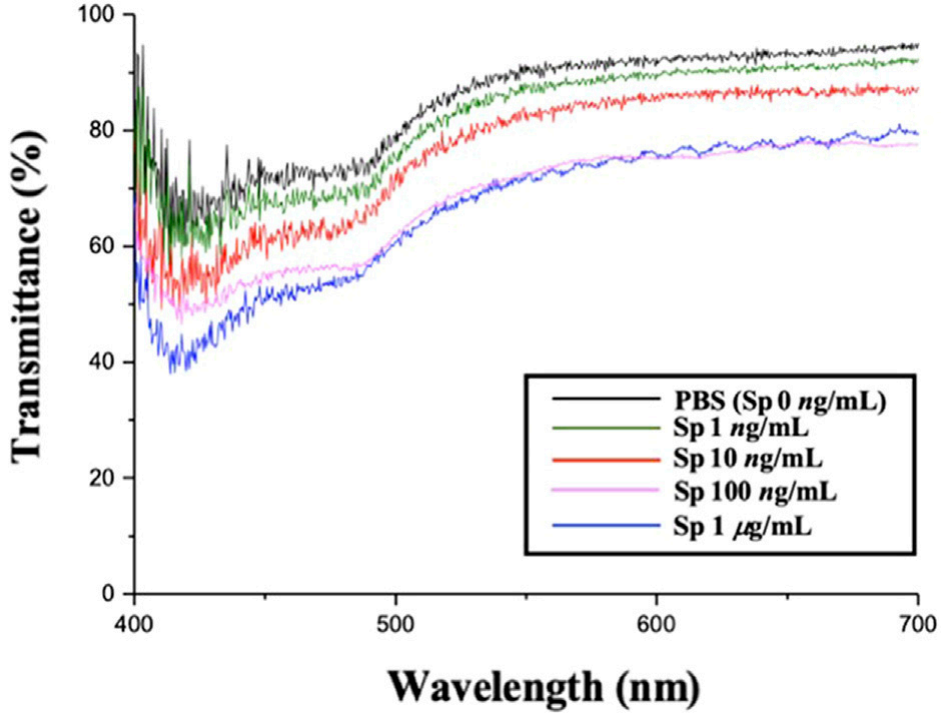
Penetration spectra of different concentrations of the spike protein detected on cholesterol liquid crystal chips.

**FIGURE 7:**
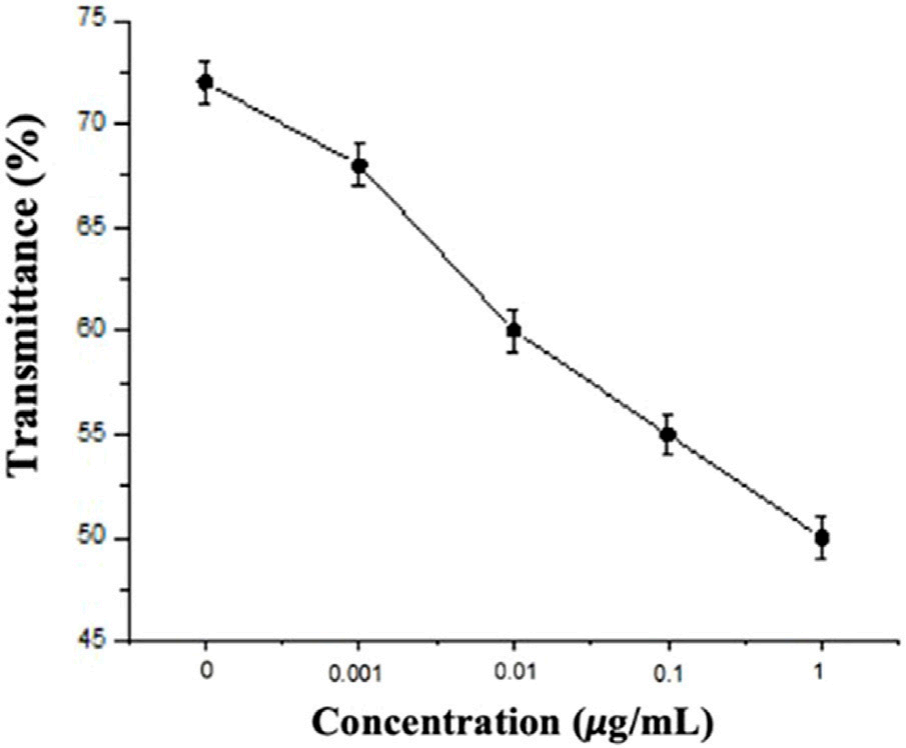
Folding line analysis of spike protein penetration spectra at different concentrations.

In this study, the experimental mechanism was established by the effect of different environments on the CLCs. Next, to investigate the sensitivity of different concentrations of Sp on the detection by the CLC wafers, four different concentrations of Sp aqueous solutions were prepared for testing, namely, 1 ng/mL, 10 ng/mL, 100 ng/mL, and 1 μg/mL, and the CLC wafers were immersed in the Sp aqueous solutions. The results were observed by taking optical images through an orthogonal polarizing microscope as shown in Figure 5, the penetration spectra of different concentrations were measured with a spectrometer in the visible wavelength band in Figure 6, and the light penetration folding analysis at the center wavelength of 450 nm with Bragg reflection bandwidth is illustrated in Figure 7. The limit of detection (LOD) of 1 ng/mL is proposed in CLC system.

Through the optical images, it can be observed that as the concentration of the Sp increased, the FC of the optical image of the CLCs gradually increased, and the texture also changed from a clean and neat texture at the beginning to a disordered texture with many FCs. The waveform of the optical spectrum also shows that the complete waveform from 0 μg/mL Sp gradually became incomplete as the concentration increased, and the light penetration also decreased. This also proved that the disturbance of the CLC molecules increased with an increase in the Sp concentration, and the CLC state changed from the P state to the FC state, so we could determine the content of the biomolecules by the texture of the optical image and the waveform and penetration of the optical spectrum.

### 3.6 Image processing and edge segmentation (Canny edge detector)

**FIGURE 8:**
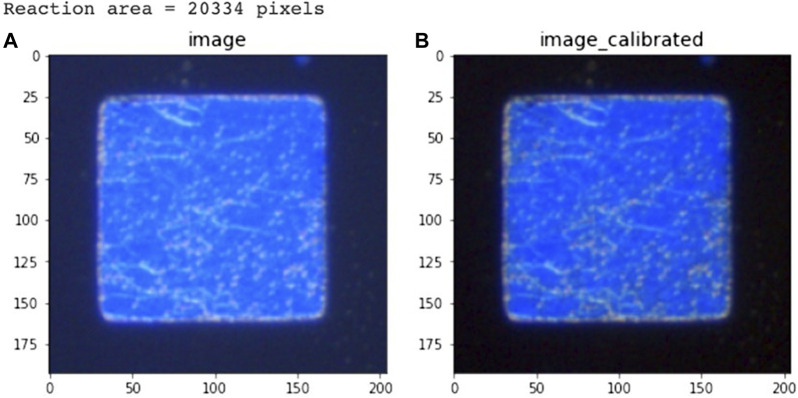
Edge detection of **(A)** the original image and **(B)** the calibrated image.

**FIGURE 9:**
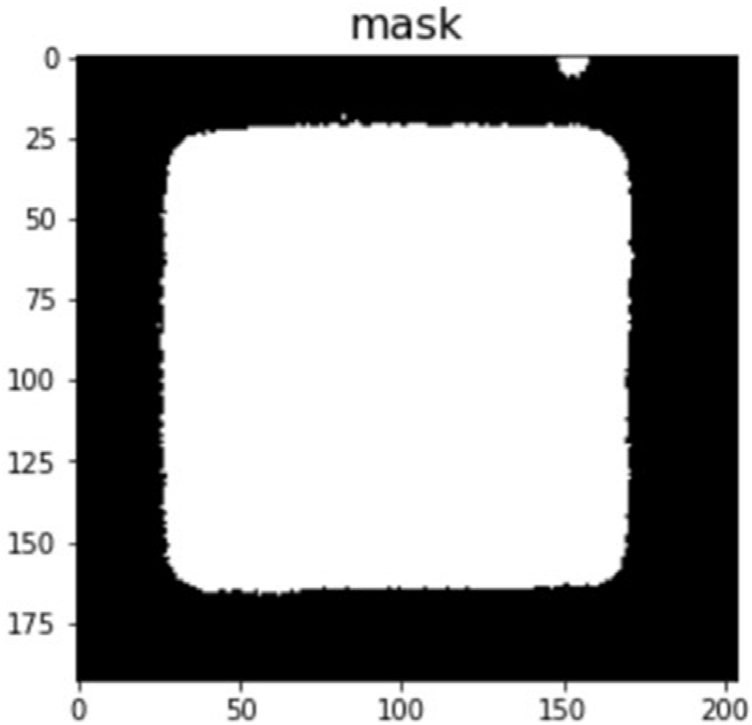
The image after masking the detection area.

**FIGURE 10:**
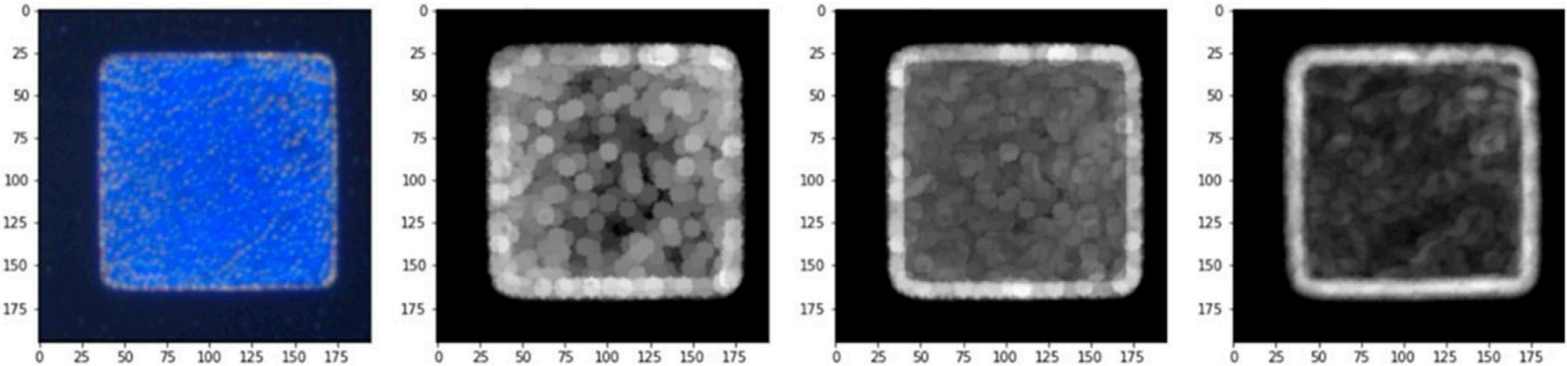
RGB gradient pixel images.

The edge segmentation technique is used in image analysis data pre-processing to capture texture features, and we used the Canny edge detector algorithm to determine the area of CLCs in the optical images. The Canny edge detector algorithm blocks, and using this image edge detection method, we could eliminate the blocks in the original image that did not need to be judged in Figure 8A, and calibrate the background value for data pre-processing to obtain Figure 8B. To eliminate the noise that cannot be handled by edge segmentation, we used a detection area mask (as shown in Figure 9) to set the initial value of the area pixel threshold to 100 and blocks smaller than 100 were considered defects and were not calculated. The mask algorithm blocks are shown and the processed image is shown in Figure 10.

### 3.7 Image color gradient analysis

The Canny edge detector algorithm was used to complete the pre-processing part of the data, and then the image gradient calculation was used as the main axis of the image analysis. The three RGB chromaticity modes were analyzed separately, the required blocks were scanned at a 5-pixel size, and the gradient change (maximum-minimum) at each point of the image was used to effectively quantify the RGB image confusion. The gradient change code is shown and the resulting image is shown in Figure 10.

### 3.8 Analysis of the confusion level

**FIGURE 11:**
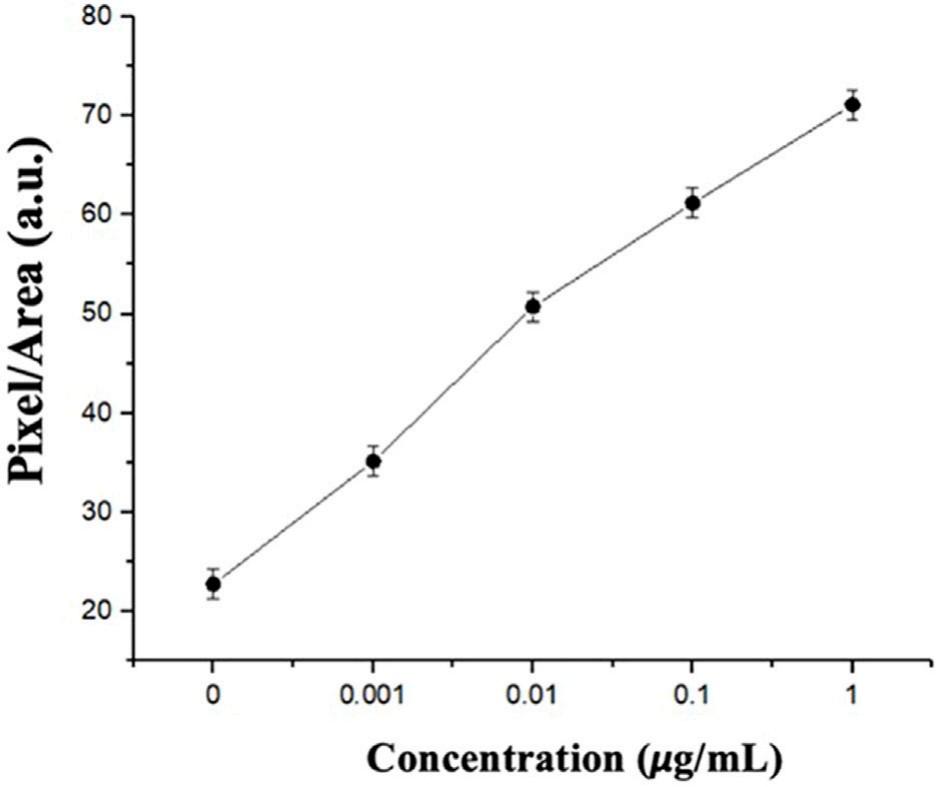
Pixel/area vs. concentrations based on the confusion analysis.

After building the algorithm to complete the CLC image analysis of the optical images, the created dataset was imported into the training model. Finally, the values of the three RGB confusion levels for all images with various echinocandin concentrations were summed and averaged to obtain the average confusion level value for each echinocandin concentration, which was the average gradient (entropy) of each pixel in the analysis area. In Figure 11, the average confusion value of each concentration is plotted as a line graph, with the *x*-axis representing the spike protein concentration and the *y*-axis representing the average confusion value. The line graph shows that the texture’s confusion gradually increased as the concentration of spike protein increased. The image recognition program quantified the degree of textural confusion of the CLCs into numerical values to demonstrate variations of the optical image texture of the CLCs.

## 4 Conclusion

For decades, LC materials have been developed in the field of bio-detectors. Compared to traditional immunosensing techniques, the advantage of using LCs is that they do not require the use of fluorescent groups or enzyme colorimetric reactions to mark biological molecules, allowing for label-free detection. We created a novel CLC detection system for aqueous environments in this study. The textural characteristics of the CLCs were digitized using a micro-optical spectrometer, image recognition software, and surface modification of the LC ligand layer to obtain a quantitative relationship between the textural and target concentrations of the CLCs. When the concentration of the spike protein increased, the optical image of the CLCs changed from a neat texture in a flat state to a chaotic texture with many focal points, the spectral penetration decreased, and the degree of chaos was successfully enumerated by image analysis of the CLC texture, and the degree of chaos increased from low to high. The system analyzed in this study not only has the advantages of being simple to operate and implement, non-marking, quantifiable, and low in cost, but it also develops a novel detection and analysis system using CLCs in conjunction with information science.

## Data Availability

The original contributions presented in the study are included in the article/supplementary material, further inquiries can be directed to the corresponding author.
